# *Cryptococcus neoformans*: Brain Preference, Gender Bias, and Interactions with *Mycobacterium tuberculosis* and *Toxoplasma gondii* in HIV-Positive Patients

**DOI:** 10.3390/microorganisms13030481

**Published:** 2025-02-21

**Authors:** Ruxandra Moroti, Adriana Hristea, Georgiana Neagu, Irina Penescu, Dragos Florea, Catalin Tiliscan, Serban Nicolae Benea

**Affiliations:** 1 Carol Davila University of Medicine and Pharmacy, 050474 Bucharest, Romania; adriana.hristea@umfcd.ro (A.H.); dragos.florea@umfcd.ro (D.F.); serban.benea@umfcd.ro (S.N.B.); 2 National Institute for Infectious Diseases Matei Bals, 021105 Bucharest, Romania; georgiana.neagu@rez.umfcd.ro; 3 Ilfov County Emergency Hospital, 022104 București, Romania; irina.penescu@yahoo.com

**Keywords:** *Cryptococcus neoformans*, *Toxoplasma gondii*, *Mycobacterium tuberculosis*, central nervous system coinfections, HIV infection, dopamine, testosterone, male sex, microbial interactions, mortality

## Abstract

*Cryptococcus neoformans*, a high-priority pathogen (WHO, 2022) and ubiquitous fungus, is responsible for hundreds of thousands of meningoencephalitis cases annually, with a high fatality rate. Its distribution is uneven: it primarily affects immunocompromised individuals (especially HIV-positive patients). Our study aims to explore the *Cryptococcus*’ brain tropism in immunosuppressed patients, its gender preference and the possible interactions with other opportunistic neurotropic microorganisms, such as *Mycobacterium tuberculosis* (MTB) and the brain microbiota, with a particular focus on *Toxoplasma gondii* (*T. gondii*). Methods: We conducted a retrospective descriptive analysis of all cases diagnosed with central nervous system cryptococcosis (Crypto-CNS) in HIV-positive patients admitted over 10 years (2010–2019) in a tertiary Romanian hospital. We examined their demographic, clinical, immunobiological, and imaging data, as well as their medical history, comorbidities, and coinfections. Results: Forty-two cases were admitted, with a male predominance (3.6:1) and a mean age of 33.3 years; 24% were diagnosed concomitantly with HIV infection and Crypto-CNS. All patients were severely immunosuppressed, with CD4 counts <200 cells/mm^3^ (median = 20.5 [1–163], mean = 31.6). Recent/concomitant tuberculosis was found in 10 (27.7%). *T. gondii*-seropositive patients developed Crypto-CNS at a lower immunological state than seronegative ones (27.1 CD4 cells/mm^3^ vs. 46.7 cells/mm^3^, means). Of 25 cases with available brain imagery, 28% had high intracranial pressure. Twelve patients (28.5%) died during the hospitalization within 26.3 days (mean, SD = 21.4); 1-year mortality increased to 50%. In-hospital mortality was associated with lower CD4 counts, increased intracranial pressure, and *T. gondii*-seropositivity. Conclusions: Crypto-CNS in HIV-positive patients mainly affects men and may be promoted by concomitant or recent tuberculosis. *T. gondii* may confer some protection even at low immune levels but increases mortality when immunity is critically low.

## 1. Introduction

*Cryptococcus* or ‘hidden-sphere’, a ubiquitous fungus present in nature (soil, trunks of many tree species, avian excreta), is annually responsive for several hundreds of thousands of meningoencephalitis, especially in HIV-infected people, with inadmissible high mortality [1]. Among the two main species complexes—*C. neoformans* species complex and *C. gatti* species complex, the first one is widespread and responsible for around 80% of global infections, almost all in immunocompromised hosts, and the second one is endemic and could severely infect immunocompetent hosts as well [2].

Unlike other yeasts, *Cryptococcus* is covered by a thick polysaccharide capsule, its hallmark, and an important virulence factor; commercial diagnosis tests identify the glucuronoxylomannan capsular antigen with very high accuracy [3,4,5]. Under the capsule is placed the cell wall, a multi-layered structure having a particular glucans composition: an abundance of 1-3 alpha-glucans and 1-6 beta glucan and a small amount of 1-3 beta glucan (thus making useless the commercial tests that detect 1-3 beta glucan in serum or other biological samples and a possible explanation for poor or no susceptibility to echinocandins) [6,7]. The presence of melanin in the cell wall confers protection against oxidative stress and antifungals [8]. It seems to play a crucial role in the dissemination of cryptococci into the central nervous system (CNS) [3,4,5], where dopamine, a substrate for melanin synthesis [9,10], is abundantly found.

Humans are infected mainly via respiratory inhalation and rarely via the skin [11]. The digestive tract is also proposed as an acquisition route [12]. Serological studies suggest that exposure to *C. neoformans* spp. complex could occur early in life (childhood) and be followed by infection and persistence even in immunocompetent hosts [13]. In immunocompromised hosts, the clinical signs could appear either after primary exposure or by reactivation, like tuberculosis (TB) [11].

From the lung (the first symptomatic/asymptomatic infection site), the fungus spreads via the bloodstream to virtually any organ, particularly the brain; the second subsequent involvement is the skin [14].

Cryptococcal meningoencephalitis is the most common and severe form [14], which appears weeks, even months after inhaling the fungi. Cryptococci cross the blood-brain barrier into the cerebrospinal fluid (CSF) and cause meningitis. Following the CSF colonization, they extend into the perivascular spaces, which become enlarged; from here, they can extend into the brain parenchyma and cause encephalitis with either miliary or nodular lesions—cryptococcomas. When the fungal burden is high, numerous fungi encased in their exuberant capsules could coalesce to form large gelatinous pseudocysts with a “soap bubble” appearance [15,16]. This last event occurs mainly in very immunosuppressed patients, whose very low immunity permits an uncontrolled flourishing of *Cryptococcus*. These coalescent masses could mechanically block the CSF flow, leading to a consequent increase in intracranial pressure and acute hydrocephalus with a generally unfavorable prognosis [16].

The diagnosis is suggested by the clinical (subacute/chronic meningoencephalitis and/or pulmonary impairment and/or skin lesions) and epidemiological aspects (immunocompromised host for *C. neoformans* or exposure in an endemic area for *C gatti*). Brain imaging is very useful for characterizing the extension of impairments. The diagnosis is certified by isolation and identification of the fungus: microscopy, cultural methods (blood, CSF, other fluids, biopsies), serologic tests (very sensitive and specific), histology, and/or molecular tools [14].

Antifungal treatment extends over a year in disseminated forms of cryptococcal disease (meningitis/CNS, pulmonary, non-CNS/non-pulmonary). It is carried out in three phases: induction (2–4–6 weeks), consolidation (8 weeks), and maintenance (12 months or until immune recovery, equivalent to secondary prophylaxis). The first-choice treatment in induction is Liposomal Amphotericin B+ Flucytosine. For consolidation and maintenance, the first-choice treatment is Fluconazole.

In the case of CNS cryptococcosis, repeated lumbar punctures for decompression are considered therapeutic procedures and are strongly indicated along with antifungal treatment and immunosuppression control. In the case of an HIV-positive patient, the antiretroviral treatment must be delayed at least 4 weeks to avoid IRIS (immune reconstitution inflammatory syndrome), which could be fatal [14,17].

Despite the combined treatment procedures, the mortality remains very high (24 to 47% at 10 weeks) [14].

Our objective was to describe Crypto-CNS in a cohort of Romanian HIV-positive patients in the most advanced stage (C3, AIDS) to explore:

(1) if there is a male gender preference for *Cryptococcus* infections in the brain of immunosuppressed patients, considering the sex-related differences, especially regarding the innate immune system, the ‘main’ defense against fungal infections
(2) the ‘reasons’ behind *Cryptococcus*’s brain tropism, given that the primary site of infection in immunosuppressed patients is established to be the brain. This includes the hypothesis of *Cryptococcus*’s need for melanin and that dopamine, a precursor to melanin, is found in large quantities in the brain. We also tried to see if there are imagistic features or other clinical or lab findings that might reflect a high burden of fungi into the CNS and that could be correlated with the mortality risk
(3) the potential interactions between *Cryptococcus* and other neurotropic opportunists frequently encountered in HIV infection, such as *Mycobacterium tuberculosis* (MTB) and *Toxoplasma gondii* (*T. gondii*), which are also formidable pathogens for HIV-infected individuals. In this regard, we tried to find out if in our HIV-infected patients who developed Crypto-CNS exists some associations with tuberculosis, as suggested by some studies in the literature [1,18,19,20,21,22], and which could be the relation between *Cryptococcus* and *T. gondii*, taking into account that the dual active infection is extremely rare (less than 10 cases reported in the whole literature since the first report, in 1986, all in HIV patients [23,24,25,26,27]), in contrast with many other frequently reported active coinfections.

In our study, we intended to fill this gap in understanding these subtle interactions among the opportunists in HIV-infected Romanian patients.

Considering our region (Romania, a Central-East European country) and our study population (HIV-infected patients), we refer to the *Cryptococcus neoformans* species complex in our analysis.

## 2. Materials and Methods

We retrospectively analyzed all the cases of CNS cryptococcal infection (cryptococcal meningitis/meningoencephalitis) in HIV-positive patients admitted to a tertiary infectious diseases hospital, National Institute for Infectious Diseases ‘Matei Bals,’ Bucharest, Romania, one of the largest infectious diseases settings in Romania, with a capacity of several hundred beds for adults and children, and the largest of the nine Romanian Regional Centers for HIV/AIDS Treatment and Care [28].

The study period was chosen to cover the 10 years up to the pandemic (including 2019). Our hospital was dedicated exclusively to COVID-19 during the pandemic, so we no longer admitted patients with other types of infections.

### 2.1. Crypto-CNS Definition

We defined Crypto-CNS as cryptococcal infections in the brain and/or meninges (meningoencephalitis or meningitis).

### 2.2. Inclusion and Exclusion Criteria

The inclusion criteria for our retrospective study were admission between 1 January 2010 and 31 December 2019 in all departments (children and adults) of our setting with Crypto-CNS and HIV-positivity. We did not have specific exclusion criteria for patients who met the inclusion criteria.

### 2.3. Data Analyzed

For each patient, we collected and analyzed demographic data (age, gender), medical history (including a previous HIV-positivity diagnosis and a TB history), clinical presentation, coinfections, comorbidities, the results of the routine blood tests and the blood cultures, and for the specific tests for un HIV-positive patient (HIV viral load, CD4 lymphocytes count, cryptococcal antigen (C-Ag), serological status for *T. gondii*, *Cytomegalovirus* (CMV), syphilis and hepatitis viruses B and C, along with cerebrospinal fluid (CSF) results (see further for details), imaging tests (chest X-ray, cerebral MRI and CT scan), therapies, evolution, outcomes, and 1-year follow-up.

### 2.4. Advanced HIV Disease Definition

World Health Organization (WHO) defines advanced HIV disease as an HIV infection with CD4 lymphocytes below 200 per cubic millimeter (mm^3^) or presenting with a WHO Stage 3 or 4 AIDS-defining illness [29].

### 2.5. Recent TB Definition

For this study, we defined ‘recent TB’ as having been diagnosed or treated for active TB within the last 36 months. The term ‘concomitant TB’ was used as a concomitant active TB.

### 2.6. Crypto-CNS Diagnosis

The Crypto-CNS diagnosis was based on the presence of *Cryptococcus* spp. (India ink, culture) and/or its antigen (C-Ag) in CSF; in case of lumbar puncture contraindication, the diagnosis relied on the presence of serum C-Ag and signs and symptoms of CNS impairment (meningitis, encephalitis), clinically and/or imagistic.

### 2.7. CSF Analysis

#### 2.7.1. Routine CSF Analysis

The routine CSF analysis performed in our labs includes several components. Quantitative cellularity is established by determining the number of nucleated cells in the CSF per mm^3^; normal values are less than five cells/mm^3^. Qualitative analyses of cells are performed when the cell count is greater than 50 cells/mm^3^ and include the percentage of mononuclear cells (lymphocytes and monocytes) and polynuclear cells (mainly neutrophils), with Giemsa smear examination used for further detail. An India ink examination is performed to detect cryptococci by highlighting their capsules. Gram and Ziehl-Neelsen staining are applied to smears of centrifuged samples to identify Gram-positive or Gram-negative bacteria and mycobacteria, respectively. By default, cultures for bacteria (standard culture media: blood agar, chocolate agar), fungi (Sabouraud agar), and mycobacteria (Loewenstein Jensen medium) are also included. The biochemical examination consists of glucose, protein, and lactic acid levels in the CSF, with normal values being greater than 40 mg/dL or greater than half of the concomitant blood glucose level for glucose, 20–60 mg/dL for protein, and less than 18 mg/dL for lactic acid, respectively.

#### 2.7.2. Other CSF Data

On request, our lab performed real-time PCR for DNA detection of MTB, *T. gondii*, and CMV in the CSF, using commercial kits (GeneXpert TB, Cepheid, CA, USA for MTB and Elitech Ingenius, ELITechGroup, Turin, Italy, for *T. gondii* and CMV, respectively).

### 2.8. Toxoplasma and CMV Serologies Interpretation

We considered *T. gondii* and CMV latent infection if we found a positive IgG serology any time before or at the moment of Crypto-CNS diagnosis.

Conversely, a negative IgG serology any time after or at the moment of Crypto-CNS diagnosis was considered an absence of infection, except an IgM-positive serology, which proved an actual or recent infection.

All other situations were interpreted as missing data.

### 2.9. Brain Imaging

Imaging examinations in our study included the standard chest X-ray and brain imaging (CT scan and MRI), with or without contrast agents. We focused on four categories of imaging impairments in the brain: nodular lesions, diffuse lesions, ventriculomegaly (hydrocephalus), and increased intracranial pressure. Signs of meningitis (meningeal enhancement) were included in the analysis when contrast agents were used. All described impairments were reported by the radiologists as such.

### 2.10. Statistical Analysis

The statistical analysis was done with IBM SPSS Statistics, version 28.0.1.0 (142).

We performed the Shapiro-Wilk test to determine whether the samples originated from normally distributed populations. Variables with a Gaussian distribution were reported as means (±standard deviation [SD]), while non-normally distributed continuous data were presented as medians (interquartile range). Differences in means between groups were analyzed using independent-sample *t*-tests, and a 95% confidence interval was calculated for the difference between population means. The Mann–Whitney test was used for non-parametric continuous variables, while categorical variables were analyzed using the Chi-square test or Fisher’s exact test to assess statistical significance.

The patients’ data were anonymized, two-tailed *p*-values were calculated, and statistical significance was defined at the 5% level.

### 2.11. Ethics Committee Approval

The Ethics Committee of the National Institute for Infectious Diseases Matei Bals approved the study. Due to its retrospective nature, informed consent was not needed.

## 3. Results

### 3.1. Demographical Data

Forty-two cases of Crypto-CNS in HIV-positive patients were admitted during the study’s 10-year period. There was a significant male predominance (3.6:1) with a median age of 30 years old [range 20–65], a mean age of 33.3 years old, and no cases under 20 years old.

### 3.2. HIV Status

All 42 study patients were classified as HIV-advanced disease. All had uncontrolled HIV infection at the time of Crypto-CNS diagnosis, with detectable HIV viral loads (VL), averaging 510,175 copies/mL [range: 64–4,760,000 copies/mL] and a median of 124,014 copies/mL. All had CD4 counts below 200 cells/mm^3^, with an average of 31.6 cells/mm^3^ and a median of 20.5 cells/mm^3^ [range: 1–163 cells/mm^3^]. Ten patients (24%) were concomitantly diagnosed with Crypto-CNS infection and HIV. The other 32 patients (76%) had previously been diagnosed with HIV but were either lost to follow-up, self-discontinued their antiretroviral medications, or were unmedicated.

### 3.3. Crypto-CNS Diagnosis

#### 3.3.1. Clinical Signs and Symptoms

At least one meningitis sign or symptom was found in 76% of patients: headache (28 patients), fever (19 patients), nuchal rigidity (17 patients), and vomiting (14 patients). Regarding encephalitis signs, 60.5% had at least one of the next: paresis (14 patients), altered mental state (11 patients), and seizures (5 patients).

#### 3.3.2. Laboratory Findings

Lumbar puncture at the Crypto-CNS diagnosis

A total of 38 (90.4%) out of 42 cases had a lumbar puncture (LP) performed at the diagnostic moment; 2 cases had LP postponed; in 2 cases, LP was not performed. Among the 38 patients with available LP results at admission, C-Ag, India ink, and fungal cultures were performed for 26 (61.9%), 32 (76.2%), and 33 (78.6%) patients, respectively (see Table 1).

At the time of Crypto-CNS diagnosis, CSF parameters showed that leucocyte counts ranged from 0 to 360 per mm^3^, with an average of 69.2/mm^3^ and lymphocyte predominance (see Table 1). CSF glucose levels averaged 41.7 mg/dL (normal values: >40 mg/dL); the mean CSF protein level was 117.6 mg/dL (normal values 20–60 mg/dL); the mean CSF lactic acid level was 28.4 mg/dL (normal values: <18 mg/dL) (Table 1).

Blood cultures were positive for *Cryptococcus* in 6 (27.2%) out of 22 patients with blood cultures available results. Serum C-Ag was positive in 16 (84.2%) patients, with 19 available results.

#### 3.3.3. CNS Imaging

Twenty-five patients underwent imaging tests (MRI or CT) with available data. An imaging test was not needed nor performed in 10 patients, and imaging data were missing for 7 patients. Abnormal findings were present in 22 out of 25 cases (88%), with many patients exhibiting multiple types of lesions concurrently: focal lesions were found in 17 patients, diffuse lesions in 14 patients, associated ventriculomegaly (hydrocephalus) in 4 patients, and raised intracranial pressure in 7 patients.

All seven patients with increased intracranial pressure were men, with a mean CD4 count of 9.7 cells/mm^3^ (compared to the general Crypto-CNS group mean of 31.6 cells/mm^3^). They all had positive CSF cultures with *Cryptococcus*, positive India ink tests, and low CSF glucose levels (38.3 mg/dL compared to 41.7 mg/dL overall in the Crypto-CNS group). All seven patients had significantly low CSF leukocyte count, with a mean of 33.1/mm^3^ vs. 69.2/mm^3^ for the entire Crypto-CNS group. Additionally, they had lower CSF protein levels, with a mean of 68.1 mg/dL vs. 117.6 mg/dL for the overall Crypto-CNS group. All these seven patients had a fatal outcome.

### 3.4. Coinfections/Comorbidities in Crypto-CNS Diagnoses Patients

In addition to the coinfections included in Table 2, 2 patients had syphilis, 1 had Kaposi sarcoma, and 14 (36.8%) of 38 with available data presented with wasting syndrome or were cachectic at the diagnosis of Crypto-CNS.

#### 3.4.1. Tuberculosis and Crypto-CNS

We had 38 patients (out of 42 in our study) with available data regarding TB status or history. Ten (26.3%) had concomitant or recent active TB (within the past 3 years, as per our statement in the Method section). An additional two patients had TB in their distant medical history (over 7 years ago). The remaining 26, with available data regarding TB status, did not have a history of TB at any time in their life.

We had two documented cases with concomitant CNS_TB: MTB and *Cryptococcus* were detected simultaneously in CSF (as in Table 1):

- A 33-year-old woman, diagnosed HIV-positive for 15 years, without HIV treatment, diagnosed, and with pulmonary TB for 2 years, nonadherent to TB treatment, presented with a CD4 count of 7 cells/mm^3^, cachectic, with fever, vomiting, nuchal rigidity, and cough; PL revealed 325 leukocytes/mm^3^, 80% neutrophils, 20% lymphocytes, glucose = 31 mg/mL, proteins = 79 mg/dL, India ink = positive, CSF culture positive for *Cryptococcus*, also CSF positive culture for MTB. She survived.
- A 43-year-old woman, concomitantly diagnosed with HIV and Crypto-CNS, presented with HIV dementia, fever, cough, wasting syndrome, CD4 count was 61/mm^3^ at admission, LP: 80 leukocytes/mm^3^, 35% neutrophils, 65% lymphocytes, glucose = 29 mg/dL, proteins = 89 mg/dL, C-Ag positive, India ink and *Cryptococcus* culture were negative, PCR-MTB positive in CSF. She survived.

The recent/concomitant TB group had a lower mean CD4 count (25.1 cells/mm^3^) at the Crypto-CNS diagnosis than the non-TB group (37.6 cells/mm^3^), but this difference did not reach statistical significance (*p* > 0.05).

Survivors in both groups (TB and non-TB) had higher mean CD4 counts vs. deceased ones (32.8 vs. 13.5, *p* = *0*.067 and 53.0 vs. 22.3, *p* = 0.042)—see Table 3.

Of 10 patients with recent/concomitant TB, 2 died during hospitalization for Crypto-CNS, and another 2 died within the following year (see Table 3). The presence or absence of recent or concomitant TB did not correlate with in-hospital or 1-year mortality.

#### 3.4.2. T. gondii and Crypto-CNS

In our series of 42 Crypto-CNS patients, there were no cases of concomitantly diagnosed *T. gondii* encephalitis and Crypto-CNS. However, a single case was diagnosed with *T. gondii* encephalitis, and 3 months later, with Crypto-CNS:

- A 38-year-old man diagnosed 6 years before with HIV infection (also with hepatitis C, being an IV drug user), who self-interrupted his antiretroviral medication, was diagnosed with cerebral toxoplasmosis: seizures, MRI with multiple lesions with ring enhancement and perilesional moderate edema, frontal and parietal on the right side, temporal and occipital in the left side; *T. gondii* PCR was positive in CSF. After 3 months, he developed new meningo-cerebral signs and symptoms: fever, vomiting, headache, nuchal rigidity, and at LP: 5 leukocytes/mm^3^, 28 mg/dL CSF glucose, 98 mg/dL CSF proteins, C-Ag positive, India ink positive, CSF cultures—positive for *Cryptococcus* spp. The diagnosis was cryptococcal meningoencephalitis. PCR for *T gondii* in CSF was not repeated. His CD4 count (immune status) at Crypto-CNS diagnosis was 24 cells/mm^3^. He initially improved and was discharged at his demand but died at a subsequent admission 2 months after the Crypto-CNS diagnosis.

We had data on 27 patients’ *T. gondii* serologic status, 11 of whom were IgG seropositive (Table 4). The only one with IgM *T. gondii* positivity was the one described above, and it was included in the *T. gondii*-seropositive group in our analyses.

*T. gondii*-seropositive patients developed Crypto-CNS at a lower CD4 mean than *T. gondii*-seronegative patients (27.1/mm^3^ vs. 46.7/mm^3^, *p* = 0.079). Moreover, *T. gondii*-seropositive patients survived at a significantly lower CD4 cell count mean than *T. gondii*-seronegative ones (26.6 vs. 52.7, *p* = 0.047). However, among *T. gondii*-seropositive patients, survivors and deceased patients had similar CD4 cell count means (26.5 vs. 28/mm^3^, respectively, *p* > 0.05). On the contrary, among *T. gondii*-seronegative patients, there were significant differences (*p* = 0.002) between the CD4 mean of survivors (with a mean of 52,7 cells/mm^3^) vs. deceased patients (with a mean of 5.0 cells/mm^3^), as shown in Table 4.

#### 3.4.3. CMV and Crypto-CNS

In our series of 42 Crypto-CNS patients, there were three cases—all men—who had concomitant CMV presence in the CSF (confirmed by CMV-DNA positive PCR) at the time of Crypto-CNS diagnosis:

- One patient with CMV-DNA detection in CSF: 27 years old, 21 CD4 count/mm^3^, was admitted with important cephalalgia and visual loss, and, along with C-Ag, India ink, and CSF culture positive, also had CMV-DNA detection; MRI showed optic neuritis and encephalitis; he survived.
- Two patients with double herpetic CMV and Epstein Barr virus (EBV) DNA detection, aged 43 and 40, had CD4 counts of 1/mm^3^ and 31/mm^3^, respectively. Both patients died within the first year following their Crypto-CNS diagnosis: one at 1 year due to cerebral lymphoma and the other at 5 months due to complications including a lung abscess, intestinal perforation requiring surgery, and a nosocomial infection.

There were 27 cases (out of 42) with known CMV serologic status, 26 of which were CMV-seropositive. There were no differences in the Crypto-CNS outcomes between CMV seronegative patients and CMV-seropositive cases.

### 3.5. The Overall Evolution of the Cases

Survival rates: After 1 month since admission (on the 30th day), 35 out of 42 patients (83.3%) survived. After 3 months (on the 90th day since diagnosis), 29 patients (69%) survived. After 6 months since diagnosis, 23 patients (54.7%) survived. At 9 months, 23 patients (54.7%) survived. After 1 year, the survival rate was 50% (21 patients survived).

Fatal outcome of Crypto-CNS: Twelve patients (28.5%) died during hospitalization—within a mean period of 26.3 days (SD = 21.4) since admission, within the first 68 days of hospitalization. At the end of the first year after diagnosis, the mortality rose to 50%—21 patients died within a mean period of 132.8 days (SD = 51.3). 

The mortality was not correlated with the patient’s age or gender.

The mortality correlated with HIV immunological status—a lower mean of CD4 count at the Crypto-CNS diagnosis (see Table 5), but not with the virological status. According to the aforementioned better immune status in survivors, we found a positive correlation between a higher level of inflammation in CSF and a higher surviving rate: the number of leukocytes in CSF: mean 105.7/mm^3^ in survivors vs. 32.7/mm^3^ in non-survivors, higher level of proteins in survivors 144.7 mg/dL mean vs. 90.6 mg/dL in non-survivors, the latest not reaching a statistical power. The fatal outcome was strongly associated with increased intracranial pressure.

We did not find a correlation between recent or concomitant TB and mortality rate.

We found a trend for *T. gondii*-seropositive patients toward fatality in the circumstances of a very low CD4 count mean in our study population. 

No statistics were performed to assess the difference in mortality for CMV, as all but one (26 out of 27) patients with available CMV status were CMV-seropositive.

## 4. Discussions

### 4.1. Gender Tropism and Possible Young Age Protective Effect in CNS Cryptococcosis

Our results are consistent with existing literature and indicate that cryptococcal CNS infection is found predominantly in males [30] and rarely affects children [30,31,32]. We observed a substantial male predominance in our Crypto-CNS cohort. The median age in our study was around 30 years old, and we did not have cases under 20 years old during the study period, even though our Institute surveyed more than one-quarter of Romanian HIV-infected patients of all ages. In Romania, there is a distinctive pattern of HIV epidemiology. We have had the largest and most homogenous cohort of patients in Europe, known as the ’Romanian cohort.’ This cohort is unfortunately linked to the so-called ’Romanian epidemiological accident’ in HIV transmission. It consists of approximately 11,000 Romanian children born between 1987 and 1990 (mostly in 1988–1989) perinatally infected, apparently accidentally, with HIV subtype F1. This subtype did not circulate in Europe in those years; Romania was an isolated ‘island’ of subtype F1, surrounded by the other European countries dominated by subtype B in the West and subtype A in the East. This cohort exhibits a male-to-female ratio of 1.2:1, as reported in 2003 when approximately 6000 children from the ‘Romanian cohort’ were already diagnosed [33].

In contrast, regions such as Asia, Africa, or other parts of the world with predominant heterosexual HIV transmission typically show a male-to-female ratio of approximately 1:3 (ranging from 1.5:1 to 1:38, the latter observed in Ugandan girls aged 15–19) [34]. Even if the male-to-female ratio of Crypto-CNS in HIV-infected populations in Asia or Africa is reported to be in some regions 1:1, it essentially reflects the same male predominance [19]

For the observed differences in age and gender predisposition to CNS cryptococcosis, we identified several possible explanations, including variances in immune defense mechanisms, sex hormone levels, and potential differences in dopamine levels in the brain (see further for details).

Differences in the immune system and those related to the influences of sex hormones provide essential support for explaining cryptococcal tropism. The immunosuppressant role of testosterone has been described in both humans and laboratory animals [35,36,37]. Moreover, macrophages isolated from females can phagocytose yeast better than macrophages isolated from males. This lowers fungal burden and increases fungal clearance [38,39]. Compared to females, peripheral blood monocytes (PBMCs) isolated from males have more significant proliferation of *C. neoformans* within them [40]. 

*Cryptococcus* has slower growth rates when isolated from females vs. males and larger capsule shedding in the presence of testosterone but not 17-β estradiol. Tucker et al. identified a growth hormone, gibberellic acid, produced in *C. neoformans* that is highly upregulated in the presence of testosterone, increasing the rate of melanization of *C. neoformans* [41].

Robust data sustain important sex differences in dopaminergic brain circuits [42,43]. Current research indicates that females have a slightly higher baseline level of dopamine in the brain than males and a different way of processing stimuli about this, such as the dopaminergic reward system [42]. Still, regarding dopamine levels strictly, one recent meta-analysis on laboratory animals reports no significant differences in basal or drug-induced extracellular dopamine activity between sexes in rodents [44].

Dopamine shows differential activity across the lifespan, with adolescence representing a period with heightened levels compared to childhood and adulthood [45], with the aging brain showing increasingly lower dopamine levels [46].

Children provide unfavorable conditions for *Cryptococcus* development (lower brain-dopamine levels and low testosterone levels), and young adult males have high testosterone levels and a relative deficit in cellular immunity vs. their female counterparts. Therefore, they are likely more prone to cryptococcal infection compared to females, who, despite their slightly higher baseline dopamine levels in the brain, are protected by more robust cellular immune responses and significantly lower testosterone levels.

Although our study aligns with existing literature regarding male predominance and also with the rarity of Crypto-CNS in pediatric cases (with no pediatric cases observed in our cohort), we did not find a statistically significant correlation between gender and increased mortality. However, it is worth noting that all seven patients in our cohort with elevated intracranial pressure (an important risk factor for mortality) experienced fatal outcomes, and all seven were male.

### 4.2. Imaging Could Reflect a High Burden of Cryptococci in CNS

Cryptococcal infection of the CNS can display three main traits [16]:

- Perivascular impairment of the interface with CSF, even CSF blockage (in case of high fungal burden): enlarged Virchow-Robin spaces, gelatinous pseudocysts (usually bilateral and symmetrical in the basal ganglia, thalami, midbrain—substantia nigra, and the periventricular white matter), hydrocephalus and intracranial increased pressure.
- Parenchymal lesions—adjacent to perivascular affected areas, above mentioned, which could have a miliary pattern (in case of higher immunosuppression) or nodular enhancing lesions—in the case of a better immune condition that permits granuloma formations).
- Meningeal enhancement—typically located at the base of the brain, often absent in the significantly immunocompromised patient (due to a low immune reactivity), avid meningeal enhancement being more likely a feature of cryptococcal immune reconstitution syndrome (IRIS).

Notably, the normal brain aspect on imagery tests does not exclude a positive Crypto-CNS diagnosis as both CT (up to 47%) and conventional MRI (up to 8%) scans may appear normal or show nonspecific findings [16]. 

In our study, imaging tests were neither considered necessary by the current physician nor performed in almost a quarter of cases. There were missing data in one-sixth of cases, and imagery data collection was inconsistent, with most examinations lacking enhancing contrast substances. Despite these limitations, we organized the results by focusing on four categories of impairments: nodular lesions, diffuse lesions, ventriculomegaly (hydrocephalus), and increased intracranial pressure. We did not consider the ’meningeal enhancement’ results in the analysis due to scarce data regarding this feature—many exams were native (without contrast agent). Our patients had proven meningitis by other methods (clinical signs and CSF analyses), and the enhancement theoretically rarely appears in very immunosuppressed patients, like those in our study. This could be interesting from the perspective of indirect evidence of CSF reactivity in the case of the disease compared to IRIS during treatment.

We have seven cases of high intracranial pressure, as we mentioned above. All seven patients had indirect proofs for a high burden of fungi in CSF: they were very immunosuppressed (a very low mean level of CD4 count in comparison with the whole study group), had positive CSF cultures, positive India ink analysis, low CSF glucose (reflecting the fungal metabolism with glucose consume) and significantly low reactivity in the CSF (a lower mean of leukocytes, comparatively with the mean of all Crypto-CNS group and a lower mean of proteins level, reflecting a poor immune-inflammatory status). 

The fatal outcome seems to be related to the accumulation of cryptococcal masses mechanically obstructing CSF flow and increasing the intracranial pressure.

All our patients with increased intracranial pressure on imaging tests died—five during hospitalization and another two during the first year after Crypto-CNS diagnosis. We found a strong correlation between the increased intracranial pressure on imaging tests and the fatal outcome in our study.

### 4.3. A Possible Symbiotic Relationship with MTB

*Cryptococcus* spp. has moved from being a relatively unknown fungal pathogen to one of the most feared opportunistic agents in immunocompromised people, especially those living with HIV. In contrast, MTB is one of the most infamous pathogens of infectious diseases. Both tuberculosis and cryptococcal disease—significantly when localized in the CNS—contribute to AIDS-related mortality. While tuberculosis is the cause of death in 33% of patients with advanced HIV infection, cryptococcosis kills another 15%. [1,18,19].

Numerous researchers have highlighted the similarities between fungal pathogens and MTB: both are intracellular agents that can survive in macrophages, both share pathways of immune response and recognition, both could act as commensals in healthy hosts with little to no symptoms while being deadly in immunocompromised hosts [47].

Sharing the same host defense mechanisms, *Cryptococcus* and MTB are prone to associate in a host whose cellular immunity is deeply affected, as is the HIV-positive patient. HIV depletes the number and functionality of immune cells, especially those responsible for type 1 immune response, exactly those mostly needed to fight against intracellular bacteria and fungi [47]; briefly, maybe too simplistic, the main action is: Th-1 cells produce cytokines, like IFN-gamma, that activate macrophages which will further engulf and kill the pathogens [48,49]. 

Moreover, MTB and *Cryptococcus* spp. can also stimulate the production of IL-10, which further impairs Th-1 pathways, leaving the host more vulnerable to subsequent infections [48,49] 

Coinfection and symbiotic relationship (biological commensalism) between *Cryptococcus* and MTB are not just theoretically explained but also reported in the literature:

√ A retrospective study published by a team from China identified 197 cases of tuberculosis/cryptococcosis coinfection in China over 46 years (between 1965 and 2016). Intriguingly, more than half (56.3%) of the coinfections are reported in just 6 years, the last ones (2010–2016) [20]. While this represents a significant increase, it is more likely due to more reliable testing and diagnosis than an actual increase in coinfections [20]. It is a heavy task for clinicians to diagnose both infections correctly and rapidly. While for cryptococcosis, the diagnosis is at reach—serum and CSF antigen (C-Ag), India ink (direct observation), and cultures (from CSF, from blood), it is more challenging to diagnose active tuberculosis in an HIV-infected patient. It is even more complicated when it comes to the diagnosis of latent tuberculosis in the same category of patients.
√ Another team from Taiwan identified 23 cases of coinfection at a university hospital between 1993 and 2006 [21].

In both articles, the authors concluded that poor outcomes might have been associated with a delay in diagnosis and treatment. There were no comments regarding an increase in the severity of any of the two infections. 

However, a recent study published in 2020 in the Journal of Clinical Medicine concluded that TB in HIV-associated cryptococcal meningitis is associated with an increased risk of death (HR = 1.75; 95% CI, 1.33–2.32; *p* < 0.001) [19]. 

The mutual support between MTB and *Cryptococcus*, theoretically explained by the microbial IL10 production that reduces the Th-1 responses, could be practically sustained by Jarvis et al. in 2010: they concluded that a history of pulmonary tuberculosis within the last 2 years was independently associated with the development of Crypto-CNS (OR 6.6; 95%CI 1.3–32.7) after adjustment for covariates including CD4 counts [22].

Romania has a very high prevalence of TB, the highest in European (EU/EEA) countries, with more than 50 cases per 100,000 population and accounting for 23.4% of all TB cases reported in 2019 as per the Annual epidemiological report by the European Centre for Disease Prevention and Control (ECDC). This percentage has been relatively stable, at least since 2015 [50]. However, TB and HIV in Romania are not largely superposed. For example, in 2019, Romania reported 11,633 TB cases to ECDC [50]. In the same year, the Romanian National HIV Register noted that 8594 TB patients were tested for HIV, and 45 were HIV positive (0.52% of Romanian TB patients were HIV coinfected) [28]. In the same register [28], in 2019, of 16,190 people living with HIV (PLWH), were recorded 506 new HIV-positive and 115 new TB-positive cases. We do not have a precise number of TB prevalence among our HIV-positive patients, but we can estimate it to be in the interval of 9% to 22.8%. We calculated (considering the year 2019) as follows: if of the 506 new HIV-positive noted patients, 45 were the TB-positive ones (of 8594 TB tested), the coinfection is 9%. However, we can consider that all 115 new TB cases belong to the 506 newly diagnosed HIV-positive individuals rather than being distributed among the 16,190 PLWH in 2019. The HIV-TB coinfection, in this case, rises to 22.8%.

In our study, up to a quarter (23.8%) of the patients presented with subsequent Crypto-CNS in 3 years of active TB or concomitantly TB. One-fifth of them (2 out of 10) had proven concomitant CNS TB infection of the Crypto-CNS diagnosis. Considering the above estimated national prevalence of TB among PLWH, if we take the upper value of 22.8%, the association of TB in our group of Crypto-CNS is no longer an exceptional fact. However, the prevalence of TB in PLWH could be lower (in the estimated interval of 9–22.8%), and in this case, a connection between *Cryptococcus* and MTB could be suggested, as these microorganisms might promote each other. We did not find higher mortality in the coinfected Crypto-CNS and TB patients—possibly because of our low number of cases.

Our study might suggest that an active, untreated TB in HIV patients could ‘prepare the terrain’ for Crypto-CNS development—which is congruent with the aforementioned literature. 

### 4.4. Dualistic Relationship with T. gondii

There are 220,000 annually reported cryptococcal meningitis in advanced AIDS (at a CD4 cell count under 100/mm^3^) globally [51] and 33% annually reactivations of cerebral toxoplasmosis in advanced AIDS patients (at a CD4 count below 50–100/mm^3^) [51].

However, concomitant *T. gondii* and Crypto-CNS infection in HIV-positive patients is very uncommon: there are seven case reports in the whole literature since 1986 [27]: the first one being reported in 1986 [23], then another two in 1987 [24,25], the following three from a series of 18 Crypto-CNS cases collected from 1988–1995 in a Spanish hospital [26] and one last case reported in 2018 [27]. This last case was the only one with an available CD4 count at the time of diagnosis, due to the timeline of HIV diagnostic tools and CD4 count availability. We also briefly described here 1 case among our 42 cases of Crypto-CNS in HIV-positive patients, but this was a subsequence of Crypto-CNS, 3 months after the cerebral toxoplasmosis diagnostic (see the Section 3, subsection *T. gondii* and Crypto-CNS). These cases are exceptional.

Could there be some antagonism between the two microbes?

**Figure 1:**
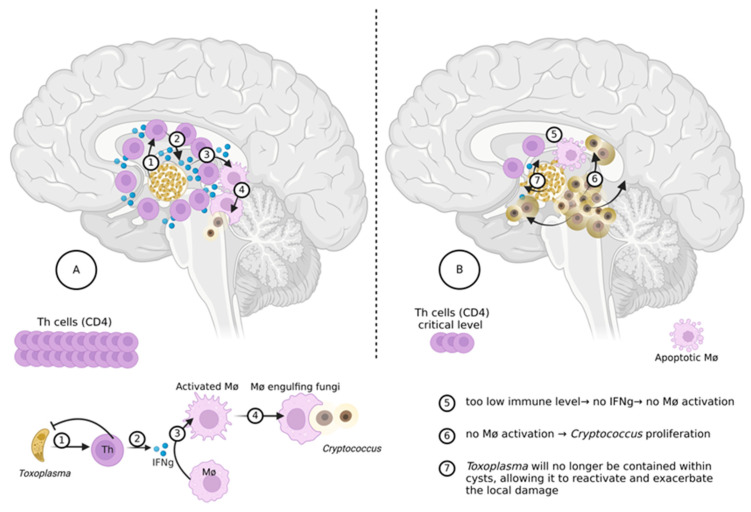
Schematical immune defense against *Cryptococcus*, in the CNS of an advanced HIV disease and latent toxoplasmosis. Legend: (**A**) The immune level > 30 (50) CD4 count/mm3. (1) *Toxoplasma* elicits Th1 responses, to be contained dormant in the cysts. (2) Th1 responses elevate local levels of gamma interferon (IFNg). (3) High local IFNg levels activate local macrophages. (4) If *Cryptococcus* enters the brain, it is destroyed by the activated macrophages. (**B**) The immune level < 50 (30) CD4 count/mm3. (5) The immune system is critically impaired, resulting in insufficient T lymphocytes to produce IFNg, even if the stimulus for this activation exists (Toxoplasma is there); consequently, local macrophages are not stimulated (activated). (6) in case of *Cryptococcus* invasion there is no defense and *Cryptococcus* flourishes. (7) Additionally, there is no containment for *Toxoplasma* which can reactivate and produce dopamine, potentially enhancing the aggressiveness of *Cryptococcus.* Created in BioRender 2025. Moroti, R. (2025) https://BioRender.com/o98i548 (Created on 9 February 2025).

On the one hand, several animal studies sustain the idea that *T. gondii* in the brain acts like a local guardian against other microorganisms that could invade the CNS, particularly *Cryptococcus*: since 1996, research publications in animals (mice) have demonstrated that brains with *T. gondii* are sheltered against cryptococcal disease [52]. The reason is the robust Th-1 type immune response elicited by *T. gondii*, with subsequent production of large amounts of IFN-gamma, a crucial cytokine for macrophage activation and adequate yeast clearance (Figure 1) [53]. 

**Figure 2:**
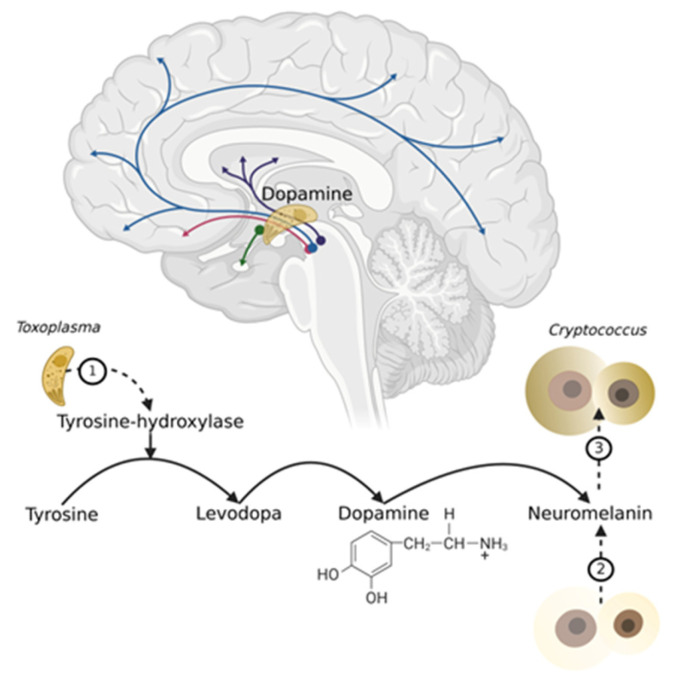
Dopamine, *Toxoplasma* and *Cryptococcus* in the Brain. Legend: (1). *T. gondii*’s tyrosine-hydroxylase contributes to dopamine synthesis (2) *Cryptococcus* reaches the brain’s neuromelanin (derived from dopamine) and undergoes melanization of the wall and capsule (3) Melanized *Cryptococcus* is protected from oxidants and proliferates exuberantly; the conglomerates of fungal bodies and their capsules could block the CSF circulation. Created in BioRender. Moroti, R. (2025) https://BioRender.com/l13l522 (Created on 9 February 2025).

On the other hand, cerebral toxoplasmosis raises the level of dopamine [54]. *T. gondii* possesses an enzyme, tyrosine-hydroxylase, that converts tyrosine to levodopa, the dopamine precursor in the brain. According to this feature, *T. gondii* infection could theoretically promote cryptococcal invasion in the brain, considering that dopamine converts to neuromelanin [55], which *Cryptococcus* uses to enhance its resistance and virulence (Figure 2). 

Thus, the presence of *T. gondii* in the brain could play a dualistic role in the relationship with cryptococcal infection, which strongly depends on the cellular immunity level. Our supposition is that the protective role predominates in regular hosts (with no or acceptable immune impairment), but this progressively diminishes with the decline in immunity. When the immunity level reaches a very low level, the balance turns to promote cryptococcal flourish. Our study supports this dualistic role of *T. gondii*: the HIV-positive patients that lacked the ‘guardian’ in their brains (*T. gondii*-seronegative) developed CNS cryptococcosis at a higher CD4 count (around 50 cells/mm^3^) than the patients that possessed the ‘guardian’ (*T. gondii*-seropositive), that kept the barrier up even at around 30 cells/mm^3^ CD4 count. However, the protection ends at a very low CD4 level when there is no immunity to be stimulated by local *Toxoplasma* presence. Based on our data, we speculate that at a CD4 level higher than 50 cells/mm^3^, *T. gondii* may confer some protection. The interval of 30–50 cells/mm^3^ CD4 is ambivalent in our research. We propose 30 cells/mm^3^ as the cut-off below which the presence of *T. gondii* becomes deleterious—likely because *T. gondii* produces dopamine supplements, thus enhancing *Cryptococcus*’s aggressivity. A limitation here is the accuracy of CD4 count measurement, which may cause variability in the proposed intervals.

### 4.5. Study Considerations

The limitation of our study, being a retrospective one over a period of 10 years, is that it consists of heterogeneous and partially missing data: the cases did not benefit from a unitary medical approach, the guidelines were not appropriately followed, many data were not registered, many were lost, and there was not a standardized imaging protocol. All these shortcomings of the study, along with a relatively small number of cases (due to the nature of this rare infection), could impair the statistical power of our findings. In this regard, we have only reported ‘trends’ for some of our conclusions. These trends, however, are primarily supported by theoretical reasons and partially by the scarce existing literature.

Future Directions

Although no definitive causal inference can be drawn, particularly regarding the ‘guardian effect’ of *T. gondii* against cryptococcal invasion in the CNS, our data generate hypotheses that merit deeper prospective investigation.

We speculate that *T. gondii*, a parasite that is part of the normal brain microbiota in 11–50% of humans, pays its price to be hosted and tolerated in our most important organ not because of the possible slight interference with the dopamine brain circuits, and possibly influencing our mood, but because of its possible role as a guardian in the brain, which is otherwise not so well-protected by our own immune system.

The next steps in our research could consist of a prospective observational multicentric about Opportunistic Neuropathogens in HIV, that includes a routine and standardized brain imaging approach, systematic documentation of *T. gondi* serologies, and also systematic search for *T. gondii*, MTB, and HIV PCRs into the CSF, and for other opportunistic neuropathogens as CMV, EBV, and JC virus, and for C-Ag and fungal cultures in serum and CSF. In addition, we could document the immunological markers in serum (along with CD4 cell count, the CD4:CD8 ratio, IFN-gamma, and IL17 levels for showing Th1 and Th17 responses in fungal defenses, possible soluble CD14 as a marker for monocytes/macrophages activation, that is crucial in defense against cryptococcal invasive infection) and in CSF (IFN-gamma, flow cytometry, along with dosing neurotransmitters as dopamine, etc.).

Another possible step is to develop an interventional study in Crypto-CNS HIV-infected patients with *T. gondii*-seropositivity, who can receive *T. gondii* therapeutic doses instead of prophylactic doses (primary prophylaxis targeting both *Pneumocystis jiroveci* and *T. gondii* reactivation is recommended for HIV-advanced disease) in case of a CD4 cell count below 30–50/mm^3^, as resulting from our study to be the cut-off for a fatal outcome.

## 5. Conclusions

CNS cryptococcosis affects primarily men and relatively spares very young ages. In this regard, we can interpret and refine WHO’S recommendations that are strong to search for serum C-Ag when the CD4 cell count is under 100/mm^3^ and are just moderate/ ‘conditional’ recommendations for CD4 count between 100 and 200/mm^3^ as that in front of a male patient is essential to systematically search for serum C-Ag even in the upper CD4 count interval. The male sex could be one of the ‘conditions’ for strong recommendation, too.

A high intracranial pressure on imaging tests (CT or MRI) may correspond with a high fungal burden in the CSF and may be linked to a fatal outcome. Active search for intracranial high pressure on imaging tests and, if found, frequently repeated LP to lower the pressure (that is, as per the international guidelines, a therapeutic measure to be done) could save lives.

Active TB in HIV-positive patients could represent a risk factor for cryptococcal infection in the next few years. The corollary is that if Crypto-CNS is diagnosed, it is important to consider and search for concomitant TB, and vice-versa—in any TB case, it is important to think about a cryptococcal coinfection, especially in advanced HIV disease. This could be practically addressed by searching C-Ag in any TB case in advanced HIV disease as a strong recommendation, even at the upper CD4 count interval of 100–200.

A positive serology for *T. gondii* could confer protection against cryptococcal infection in the CNS in case of an acceptable CD4 count. Still, a very low level of immunity could contribute to an unfavorable outcome. A systematic search for *T. gondii* serologic status in HIV infection is mandatory as per the guidelines. It is also important to correlate *T. gondii* serologic status with the CD4 count in any HIV-infected patient. It is worth systematically searching for serum C-Ag in advanced HIV disease (as per the guidelines), regardless of CD4 count in the case of positive *T. gondii* serology, because Crypto-CNS could manifest clinically at a lower CD4 count than in *T. gondii*-seronegative patients. By doing so, we could detect cases before they become clinically manifest and influence the outcome. Given the potential for a fatal outcome in cases of Crypto-CNS with *T. gondii* positive serology and a CD4 count lower than 50 cells/mm^3^ (particularly below 30 cells/mm^3^), we might consider *T. gondii* treatment instead of primary prophylaxis, although this remains speculative.

## Figures and Tables

**Table 1:** CSF findings in patients with Crypto-CNS.

Leuko/mm^3^ Mean (SD) N = 36	% Ly Mean (SD) N = 12	Glu Mean (SD) mg/dL N = 38	Prot Mean (SD) mg/dL N = 38	Lactic Acid Mean (SD) mg/dL N = 31	C-Ag (+) of Total Available Results (%) N = 26	India Ink (+) of Total Available Results (%) N = 32	Culture (+) of Total Available Results (%) N = 33	Other Bacteria Concomitantly Found in CSF
69.2 (105.2)	71.5 (31.5)	41.7 (19.8)	117.6 (116.1)	28.4 (13.6)	24 (92.3%)	25 (78.1%)	25 (75.7%)	2 cases MTB

Legend: Leuko = leukocytes (normal values in CSF < 5 cells/mm^3^); the value is expressed as mean and standard deviation (SD); N = number of cases with available results; Ly = Lymphocytes (the percentage of Ly was performed when the leukocytes count is greater than 50 cells/mm^3^, meaning for 12 cases out of 36 with available data); Glu = glucose; normal values for glucose in CSF: >40 mg/dL or >1/2 of concomitant glycemia; Prot = proteins; normal values for proteins in CSF: 20–60 mg/dL; normal values for lactic acid in CSF: <18 mg/dL; The values for Glu, Prot, and lactic acid are expressed as mean and standard deviation (SD); C-Ag = cryptococcal antigen; The results for C-Ag positivity, positive India ink tests, and positive cultures for *Cryptococcus* are expressed as both the number of positive samples out of the total samples performed and as the percentage of the total samples performed, in the brackets. MTB = *Mycobacterium tuberculosis*. MTB was detected in the CSF of two patients, along with *Cryptococcus* and HIV. No other bacteria were found in the CSF.

**Table 2:** Active or latent coinfections in patients with Crypto-CNS.

Coinfection	Number of pts Screened or with Available Data N (%)	Number of pts with Confirmed Coinfection N (%)
TB (Recent/Concomitant)	36 (85.7)	10 (27.7)
*T. gondii* Latent infection	27 (64.2)	11 (40.7)
CMV Latent infection	27 (64.2)	26 (96.3)
Hepatitis B or C	35 (83.3)	10 (28.6)

Legend: Latent infection = IgG-positive serology (for *T. gondii* or CMV), pts = patients; N = number of patients.

**Table 3:** **TB coinfection**: CD4 count at the Crypto-CNS diagnosis and the in-hospital mortality CD4 count related.

TB	CD4 Cell Count at Crypto-CNS Dx Mean	Number of In-Hospital Deaths (%)	CD4 Cell Count of the Survivors’ Group Mean	CD4 Cell Count of the Deceased’s Group Mean	*p*-Value for CD4 Difference Survivors vs. Deceased
Concomitant/recent TB N = 10	25.1	2 (20)	32.8	13.5	**0.067**
No TB history N = 26	37.6	8 (30.8)	53.0	22.3	**0.042**

Legend: pts = patients; recent TB = TB in the previous 3 years; No TB history = the patients who did not have a known history of TB at any time in their life *p*-value of the difference in CD4 cell count means between survivors and deceased.

**Table 4:** **Latent toxoplasmosis:** CD4 count at the Crypto-CNS diagnosis and the in-hospital mortality CD4 count related.

*T. gondii* Serology (IgG)	CD4 Cell Count at Crypto-CNS Dx Mean	Number of In-Hospital Deaths (%)	CD4 Cell Count of the Survivors’ Group Mean	CD4 Cell Count of the Deceased’s Group Mean	*p*-Value for CD4 Difference Survivors vs. Deceased
*T. gondii* IgG (+) N = 11	27.1	5 (45.4)	26.5	28.0	>0.05
*T. gondii* IgG (−) N = 16	46.7	2 (12.5)	52.7	5.0	**0.002**
*p*-value *	**0.079**	**0.084**	**0.047**	**0.067**	

Legend: pts = patients; *p*-value of the difference in CD4 cell count means between survivors and deceased; *p*-value * of the difference between *T. gondii* seropositive and seronegative patients about mean of CD4 cell count at diagnosis (Dx); mean of CD4 cell count among survivors and deceased and about the number of in-hospital deaths.

**Table 5:** Correlation of Crypto-CNS mortality with immune status, with clinical manifest signs and symptoms, with inflammation level/reactivity in CSF (number of leukocytes per cubic mm), and with the increased intracranial pressure.

Characteristics	Survival vs. In-Hospital Death	*p*-Value	Survival vs. Late Death	*p*-Value
CD4 count/mm^3^ (means)	33.7 vs. 26.4	0.58	43.2 vs. 20.1	**0.052**
CSF leukocytes/mm^3^ (mean)	78.8 vs. 40.5	0.35	105.7 vs. 32.7	**0.035**
Increased intracranial pressure (on imaging)	2 vs. 5	**0.001**	0 vs. 7	**0.001**
*T. gondii* IgG (+)	6 vs. 5	**0.084**	4 vs. 7	**0.061**

Legend: IgG (+) = *T. gondii* IgG-positive; In-hospital death = death during hospitalization; Late death = death in the first year; NS = non-statistically significant (*p* > 0.05).

## Data Availability

The data presented in this article are available upon request from the corresponding authors. The raw patients’ data are preserved in the electronic database of the National Institute for Infectious Diseases Matei Bals, Bucharest, Romania, and consist of their files (diagnosis, history, outcome, and all laboratory tests). Our Institute database contains the patients’ data since 2000.
